# Gene polymorphisms in *APOE, NOS3*, and *LIPC *genes may be risk factors for cardiac adverse events after primary CABG

**DOI:** 10.1186/1749-8090-4-46

**Published:** 2009-08-19

**Authors:** Sandra Eifert, Astrid Rasch, Andres Beiras-Fernandez, Georg Nollert, Bruno Reichart, Peter Lohse

**Affiliations:** 1 Department of Cardiac Surgery, Ludwig Maximilians University Munich, Germany; 2 Department of Clinical Chemistry, Ludwig Maximilians University Munich, Germany

## Abstract

**Introduction:**

Coronary artery disease progression after primary coronary artery bypass grafting may, beside classical atherosclerosis risk factors, be depending on genetic predisposition.

**Methods:**

We investigated 192 CABG patients (18% female, age: 60.9 ± 7.4 years). Clinically cardiac adverse events were defined as need for reoperation (n = 88; 46%), reintervention (n = 58; 30%), or angina (n = 89; 46%). Mean follow-up time measured 10.1 ± 5.1 years. Gene polymorphisms (***ApoE, NOS3, LIPC, CETP, SERPINE-1, Prothrombin***) were investigated separately and combined (gene risk profile).

**Results:**

Among classical risk factors, arterial hypertension and hypercholesterinemia significantly influenced CAD progression. Single ***ApoE, NOS3 ***and ***LIPC ***polymorphisms provided limited information. Patients missing the most common ***ApoE ***ε3 allele (5,2%), showed recurrent symptoms (p = 0,077) and had more frequently reintervention (p = 0,001). ***NOS3 ***a allele was associated with a significant increase for reintervention (p = 0,041) and recurrent symptoms (p = 0,042).

Homozygous ***LIPC ***patients had a higher reoperation rate (p = 0.049).

A gene risk profile enabled us to discriminate between faster and slower occurrence of cardiac adverse events (p = 0.0012).

**Conclusion:**

Single ***APOE, LIPC ***and ***NOS3 ***polymorphisms permitted limited prognosis of cardiac adverse events in patients after CABG. Risk profile, in contrast, allowed for risk stratification.

## Background

Coronary artery disease (CAD) is a multifactorial disorder, accounts for roughly one-half of all cardiovascular deaths, and is a major cause of morbidity and mortality. Classical risk factors for CAD such as smoking or alterations in lipid metabolism are well known for decades to increase the incidence [1,2]. Patient counselling and medical therapy of risk factors have become the basis for secondary CAD prevention after primary coronary artery bypass grafting (CABG). Appearance of cardiac adverse events after primary CABG is frequent and leads to recurrent angina, myocardial infarction, and the need for reintervention.

Apolipoproteins play a major role in lipid metabolism. They transfer water insoluble lipids in their soluble state and enable lipid transport mechanisms. Furthermore, they may act as ligands for lipid receptors. Apolipoprotein E (ApoE) is a ligand for the low density lipoprotein (LDL) receptor and regulates catabolism of lipoproteins. ApoE is also the main protein component of the very low density lipoproteins (VLDL) and high density lipoproteins (HDL). ***ApoE ***polymorphisms generate more than 10 percent of the interindividual difference of plasma cholesterol. There are several forms of ApoE. Among them, ApoE4 has a higher and ApoE2 much lower affinity to the LDL receptor. That is the reason, why lipoproteins of ε4 carriers disappear much faster from plasma. Consecutively, that leads to downregulation of hepatic LDL receptor, resulting in rise of plasma LDL cholesterol. Therefore, ApoE4 may potentially be considered atherogenic, while ApoE2 seems to show a protective effect. That explains the higher cardiovascular risk of ε4 carriers [3]. Baroni et al., demonstrated correlation between ApoE4 polymorphism and the incidence of CAD [4].

Dysfunction of the vascular endothelium, defined as impaired nitric oxide (NO) activity, may also play a substantial role in the initiation and progression of atherosclerosis [5,6]. Most important in this regard appears to be activity or quantity of the enzyme endothelial nitric oxide synthase (eNOS). Several single nucleotide polymorphisms (SNPs) have been described in the ***NOS3 ***gene and some of them have been associated with cardiovascular diseases such as 786T/C and 894G/T polymorphism. The 786 CC allele is connected to a reduced gene transcription and probably connected to a decreased NO production. C allele seems to be associated to a higher atherosclerotic risk and coronary spasm [6]. Wang et al. could demonstrate a significantly higher incidence of the rare homozygous eNOS 4a allele in patients with significantly stenosed peripheral arteries. If 894T allele is present in 894G/T polymorphism eNOS activity may be impaired [5]. This polymorphism may also influence NO release of thrombocytes. We investigated the insertion/deletion polymorphism in intron 4. It is located on chromosome 7q36.

Hepatic lipase (LIPC) is a lipolytic enzyme synthesized in hepatocytes playing a major role in HDL metabolism. It takes part in hydrolysis of triacylglycerides and phospholipids of HDL_2 _into antiatherogenic, cholesterol rich HDL_3 _as well as catalysation of hydrolysis of big triacylglyceride rich LDL into small, compact, and atherogenous LDL particles. There is a positive correlation between concentration of small, compact LDL and LIPC activity [7].

The ***LIPC ***gene is located on chromosome 15 (q21-q23). De Andrade et al., showed a significant correlation between male carriers of a *LIPC *polymorphism and a higher CAD risk independently of conventional risk factors. The C202G polymorphism may also be associated with higher triglyceride and lower HDL levels [7].

The influence of the classical risk factors and of genetic polymorphisms, whose protein products play a role in lipid metabolism, coagulation, and nitric oxide metabolism, on the appearance of cardiac adverse events in patients after CABG, who receive contemporary medical treatment, is still unknown.

We hypothesized that CAD risk factors, perioperative parameters, and a genetic predisposition determine the occurrence of adverse cardiac events after primary CABG in individual patients. The hypothesis was tested by retrospectively investigating a group of patients, which underwent primary CABG at our institution more than five years ago. Genetic polymorphisms known to be risk factors for CAD were determined in this patient cohort. Reoperations, reinterventions, and angina at follow-up served as clinically relevant cardiac adverse events.

## Methods

### Patients

One-hundred-and-ninety two patients, who underwent their first isolated CABG between 1979 and 1999, were investigated. Demographic data and medical therapies at the time of prior CABG are listed in Table 1. All patients made a follow-up visit at our Department between March and October 2007. After written informed consent was given, EDTA blood was drawn for genetic analyses. Reoperation for CAD, reintervention (PTCA and/or stenting and/or hospital admission for myocardial infarction), angina at the time of follow-up, and a combined endpoint of the three previous ones, further referred to as recurrent symptoms were defined as cardiac adverse events. Most patients (n = 137; 71%) had either a second CABG (n = 88; 46%; 9.3 ± 3.3 years p.o.), a reintervention (n = 58; 30%; 10.4 ± 5.9 years p.o.), or suffered from an angina at follow-up (n = 89; 46%; 12.6 ± 5.8 years p.o). All patients received medical therapy at follow-up. Aspirin (87%), beta blockers (70%), statins (50%), ACE inhibitors (35%), and calcium antagonists (24%) were most commonly prescribed. Patients with progression of CAD were more likely to receive intensified therapy with ACE inhibitors (p = 0.04), and beta blockers (p = 0.008).

**Table 1:** Demographics of patients and medical therapy at the time of primary CABG

Patients [#]	192
Female [#]	34 (18%)

Age [Years]	60.9 ± 7.4

Ejection fraction [%]	63.0 ± 14.5

NYHA Class	3.0 ± 1.0

Bypasses [#]	2.4 ± 1.0

Left internal thoracic artery grafts [#]	1.0 ± 0.5

Saphenous Vein Grafts [#]	1.5 ± 1.0

Additional Grafts [#]	0.4 ± 0.5

Aspirine Intake [%]	75

Beta blockers [%]	70

Statins [%]	50

ACE Inhibitors [%]	35

Calcium Channel Blockers [%]	24

### Genetic analyses

Polymorphisms in genes coding for factors involved in lipid metabolism: apolipoprotein E (***APOE***) [4,8], hepatic lipase (***LIPC***) [7], and cholesteryl ester transfer protein (*CETP*) [9], the NO-donor system: endothelial NO synthase (***NOS3***) [6], and the coagulation system: plasminogen activator inhibitor 1 (***SERPINE-1***) [10], coagulation factor V (*F5*) [11], and coagulation factor II (prothrombin (*F2*)) [12] previously described to play a role in the development of CAD were chosen. The genes investigated, their polymorphisms, function as well as the primer sequences are shown in Additional File 1.

### Restriction Fragment Length Polymorphism (RFLP) Analysis

To investigate the selected seven polymorphisms, restriction fragment length polymorphism (RFLP) analysis was used. The patients genomic DNA was isolated from a 200-μl aliquot by means of the QIAamp blood mini kit (Qiagen, Hilden, Germany). The PCR reactions contained 5 μl PCR buffer (ABgene, Hamburg, Germany), 5 μl dNTPMix (Fermentas Life Sciences, St. Leon-Rot, Germany) as well as 1 μl forward and 1 μl backward primer (400 nmol; Thermo Electron Corporation, Ulm, Germany) in a volume of 50 μl containing about 150–200 ng of genomic DNA. Depending on the specific polymorphism, the PCR was comprised of an initial denaturation step (15 minutes at 95°C), 35 to 40 cycles of 95°C for 20 to 30 seconds, 58 to 72°C for 20 to 30 seconds, and of 72°C for 30 sec).

The restriction digests were performed in a 10-μl volume containing the restriction enzyme (Fermentas Life Sciences, St. Leon-Rot, and New England BioLabs, Frankfurt am Main, Germany), 1× restriction buffer, and the PCR product. The digests were incubated overnight at 37°C and analysed by electrophoresis in a 1,5–3% agarose gel which was subsequently stained with ethidium bromide. Details of the polymorphisms analyzed are summarized in Additional File 1.

### Risk profile

Because SNPs had a relatively low prevalence in our limited patient cohort and CAD is a multifactorial disease, we constructed a risk profile. Carriers of the risk profile were defined as having one of the following polymorphisms: hetero- or homozygous for the variants of ***eNOS 4ab***, the ***ApoE ***allele combination 2/4 or 4/4, homozygous expression of ***LIPC ****variant*, homozygous expression of the ***CETP ***variant, and hetero- or homozygosity for the ***prothrombin ***G20210A variant. Patients had to be homozygous for *PAI-1 *5G insertion.

### Statistics

For statistical data analysis Microsoft^® ^EXCEL 2002 and SPSS for Windows (Version 12.0, SPSS Inc., Chicago, IL, USA) were used. The primers for the PCR analyses were designed with the help of the computer program "PrimerExpress" (Applied Biosystems).

Values are expressed as mean and standard deviation of mean. The Student's t-Test was used to compare absolute quantitative values. Angina was censored at the day of follow-up, because a precise onset of angina could not be determined. Freedom from reoperation, reintervention, and the combined endpoints (reoperation, reintervention, or angina at follow-up) were calculated by the actuarial method and tested with the log-rank test. Every univariate parameter reaching or approaching significance (p < 0.2) was then tested in a Cox multivariate model using the conditional backward method. P < 0.05 was considered significant.

## Results and discussion

### Results

#### Preoperative risk factors of CAD and occurrence of cardiac adverse events

Documented risk factors at the time of primary CABG had limited impact on the *occurrence of cardiac adverse events*. Arterial Hypertension was evident at the time of primary surgery in 73% of our patients and did significantly increase the risk of progression in the sense of reintervention (p = 0.04). Eighty two percent of our patients showed hypercholesterinemia at primary CABG. It tended (p = 0.073) to increase the risk of recurrent symptoms. Just 17% of the investigated CABG patients suffered from diabetes. Significant results are shown in Table 2.

**Table 2:** Preoperative arterial hypertension and hypercholesterinemia in correlation to occurrence of cardiac adverse events

				**Freedom from [%] at **			
				
**Factor**	**Definition**	**n **	**Outcome**	**5 years**	**10 years**	**15 years**	**p**
**Hypertension**	*Yes*	140	*Reintervention*	90.6	81.7	66.4	0.04
	*No*	52		95.2	88.9	88.9	

**Hypercholesterinemia**	Yes	159	Combined	84.4	46.2	10.0	0.073
	No	33		96.7	76.7	18.5	

Significant differences are in italics (p < 0.05) print.

#### Perioperative parameters and occurrence of cardiac adverse events (Table 3)

**Table 3:** Perioperative parameters and occurrence of cardiac adverse events

				Freedom from [%] at			
Factor	Definition	n	Outcome	5 years	10 years	15 years	p

Age	< 60 years	110	Reoperation	95.3	65.7	19.9	0.052
	> 60 years	82		93.8	78.7	46.3	

	< 60 years	110	Reintervtion	93.2	88.6	77.4	0.15
	> 60 years	82		90.8	79.1	58.1	

# of bypasses	**< 3**	**110**	**Reoperation**	**91.6**	**61.8**	**24.8**	**0.005**
	**≥ 3**	**82**		**98.7**	**81.6**	**22.2**	

Significant differences are in italics (p < 0.05) or bold (p < 0.01) print.

Perioperative parameters demonstrate selection bias for future therapy. Elderly patients (>60 years at primary CABG) were less likely to undergo reoperation (p = 0.05), but tended to be more often selected for reinterventions (p = 0.15). Moreover, patients with 3 or more bypasses had less reoperations than patients with 2 or less (p = 0.005).

#### Gene polymorphisms and occurrence of cardiac adverse events (Table 4)

**Table 4:** Genetic Polymorphisms and occurrence of cardiac adverse events

				Freedom from [%] at			
Factor	Definition	n	Outcome	5 years	10 years	15 years	P
***eNos ***	*bb*	141	*Reintervention*	93.9	88.7	75.9	0.041
***4ab***	*aa/ab*	51		87.5	67.9	62.7	

	*bb*	141	*Combined*	89.3	56.0	13.2	0.042
	*aa/ab*	51		80.4	40.6	5.1	

***ApoE***	**2 3/3 3/3 4**	**182**	**Reintervention**	**92.3**	**84.5**	**75.1**	**0.001**
	**2 4/4 4**	**10**		**90.0**	**64.3**	**32.1**	

***LIPC***	*WW/WM*	144	*Reoperation*	96.4	74.6	27.5	0.049
	*MM*	48		89.5	54.5	9.9	

See *Additional File *1for abbreviations of polymorphisms. M: Mutation; W: Wildtype. Significant differences are in italics (p < 0.05) or bold (p < 0.01) print.

Out of seven polymorphisms, the following single gene polymorphisms showed significant results and therefore, will results will be restricted to these single polymorphisms: Mutation of ***eNOS ***increased the risk for reinterventions (0.041) and for recurrent symptoms (p = 0.042). A similar effect on reinterventions (0.001) and recurrent symptoms (0.077) was observed in patients missing the most common type 3 allele of *ApoE*. Patients homozygous for the *LIPC *mutation had a higher incidence of reoperations (p = 0.049).

#### Distribution of the three ApoE alleles

*Detailed results of the ApoE alleles are demonstrated in *Table 5 Patients, who miss the most common allele ε3 of ***ApoE ***(5.2%), showed recurrent symptoms (p = 0.077) and had more frequently to undergo a reintervention (p = 0.001).

**Table 5:** Distribution of the three ApoE alleles

***ApoE***	**ε2/2**	**ε2/3**	**ε3/3**	**ε3/4**	**ε4/4**	**ε4/2**	Total
Number[n]	0	22	114	46	2	8	192

Percentage[%]	0	11,5	59,4	24,0	1,0	4,2	100

Patients, who miss the most common allele ε3 of ***ApoE ***(5,2%), showed recurrent symptoms (p = 0,077) and had more frequently to undergo a reintervention (p = 0,001).

#### Distribution of eNOS 4ab polymorphism

The homozygous genotype bb (wild type = insertion/insertion) of ***eNOS 4ab ***insertions/deletion polymorphisms was found in 73.4%. The rare allele a with deletion showed a level of 26.6% of patients, but only 1.6% had homozygous genotype aa (deletion/deletion) and 25% had heterozygous genotype ab (deletion/insertion). Results are depicted in Table 6.

**Table 6:** Distribution of eNOS 4ab polymorphism

***eNOS***	**bb**	**ab**	**aa**	Total
Number[n]	141	48	3	192

Percentage[%]	73,4	25,0	1,6	100

The homozygous genotype bb (wild type = insertion/insertion) of ***eNOS 4ab ***insertions/deletion polymorphisms was found in 73,4%. The rare allele a with deletion showed a level of 26,6% of patients, but only 1,6% had homozygous genotype aa (deletion/deletion) and 25% had heterozygous genotype ab (deletion/insertion).

The a allele of ***NOS3 ***was associated with a significantly risk increase of reintervention (p = 0.041) and recurrent symptoms (p = 0.042). Only 5.1% of patients with allel a, were free of symptoms after 15 years. Carrier of heterozygous expression had a 30% higher likelihood of reoperation, meanwhile patients with homocygous expression were 30% more likely, not to undergo a reoperation (p = 0,04).

#### Distribution of LIPC genotypes in regard to C→G exchange at codon 202

Analyses showed a higher incidence of reoperation in patients with homozygous genotype (p = 0.049). Table 7 shows the distribution of LIPC genotypes in our patient's population.

**Table 7:** Distribution of LIPC genotypes in regard to C to G exchange

***LIPC***	**CC**	**CG**	**GG**	**Total**
Number[n]	49	95	48	192

Percentage[%]	25,5	49,5	25,0	100

The homozygous genotype bb (wild type = insertion/insertion) of ***eNOS 4ab ***insertions/deletion polymorphisms was found in 73,4%. The rare allele a with deletion showed a level of 26,6% of patients, but only 1,6% had homozygous genotype aa (deletion/deletion) and 25% had heterozygous genotype ab (deletion/insertion).

#### Risk profile and occurrence of cardiac adverse events (Figure 1)

**Figure 1:**
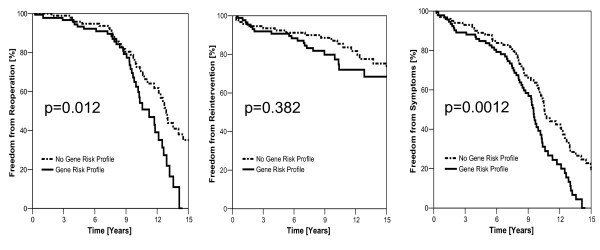
**Risk profile and occurrence of cardiac adverse events**. Out of the seven investigated gene polymorphisms, six were combined for a risk profile (see text). Patients with the risk profile had significantly more reoperations (0.012) and were more likely to have recurrent symptoms (0.0012). The incidence of interventions (PTCA, stent) were not different among the groups (p = 0.38). However, multivariate Cox regression analysis revealed that only the risk profile had significant impact on the incidence of cardiac adverse events (p = 0.004).

Patients with the risk profile had significantly more reoperations (0.012) and were more likely to have recurrent symptoms (0.0012). The incidence of percutaneous interventions were not different among groups (p = 0.38). However, multivariate Cox regression analysis revealed that only the risk profile had significant impact on the progression of CAD (p = 0.004).

## Discussion

In our study, we investigated a subset of gene polymorphisms known from other investigations as suspects in the development of CAD. Occurrence of cardiac adverse events after primary CABG was significantly influenced by genetic polymorphisms; among the seven evaluated polymorphisms ***ApoE ***and ***eNos ***variants had the highest impact on reoperations and reinterventions.

### ApoE Polymorphism

Several studies focused on the effects of ***ApoE ***after cardiopulmonary bypass and reported a higher incidence of inflammation [8] and nephropathy [13] in patients with the E4 allele; depending on the trial more or less neurological sequelae were seen with the E2 allele [14,15]. In Kuukasjärvi et al. [16] investigated the ApoE E4 allele and found, in contrast to most of the studies, this polymorphism not to be a predictor for reoperation. Interestingly, in our study patients without the most frequent allele E3, thus only having the risk alleles E2 and E4, had a more aggressive type of CAD. In regard to the total population, there is no other gene product, which has such a big influence on the individual cholesterin level such as ***ApoE***. Especially the ε4 allele is increasing the plasma level of LDL. In various investigations ***ApoE ***was associated with a higher incidence of atherosclerosis. Baroni et al conducted a study with 6 polymorphisms influencing the lipid system (*ApoE, ApoAI, ApoCIII, ApoB, lipoprotein lipase LPL und LIPC *[4]. One hundred and two patients with diagnosed CAD were enrolled and 104 healthy patients served as controls. Significant difference between CAD and healthy patients could be demonstrated for Apolipoprotein E, ApoB and HL. ApoE ε4 allele was associated with a manifest CAD. Results of our study were similar: patients with deletion of the most common allele ε3 that means they carry the rare combination of either ε2/4 or ε4/4 (isoform ε2/2 is extremely rare) had more frequently to undergo a reintervention. After incidence is so low (2–3% of general population), evaluation is limited. Therefore, a higher number of patients would be desirable.

### eNOS 4ab

The investigated ***eNOS ***variant has been associated with a higher susceptibility to coronary lesions in smokers and NO metabolites were 20% decreased in patients with the 4aa variant [5]. For endothelial NOS at least 4 frequent polymorphisms (G894T, Glu298Asp, T786C, and the one used in this study) are well established and described to be risk factors for CAD. The argument of having no specific hypothesis to investigate just one and not all other polymorphisms is valid. Further more, some authors see differences in subgroups, i.e. smokers vs. non smokers and young adults. These oppositional results are frequently seen in genetic studies and may be due to the multi factorial origin of the disease. For instance, in japanese and caucasians similar patterns of ***eNOS ***alleles were observed [17], but in afroamericans the incidence is generally much lower. Especially environmental factors are difficult to record such as smoking, which is particularly important in eNOS. In our study, the a allele of ***eNOS 4ab ***polymorphism was associated with a significantly higher risk for reintervention and recurrent symptoms.

### LIPC C202G

Enzyme activity of hepatic lipase plays a major role in regulating the lipid metabolism. In the above mentioned study from Baroni et al. in carriers of the g allele of C202G mutation, a decreased level of HDL cholesterol and increased level of triglycerides was observed [4]. Interestingly, the authors found another independent factor regarding clinical endpoints: carrier of homo- or heterozygous g allele had significantly more frequently a second adverse event. Also our results demonstrate a similar course: patients with homozygous expression of the g allele (n = 48) needed more frequently a reoperation. For this mutation, Murtomaki et al, demonstrated a binding imbalance towards additional *LIPC *polymorphisms such as L334F, T457T and C480T). Last is related to a low LIPC activity in CAD patients [18]. That means that C202G mutation is a simple marker for additional LIPC polymorphisms and their mutations.

Recently Taylor and associates [19] reported on the influence of lipoprotein lipase locus on the progression of atherosclerosis in coronary artery bypass grafts and identified the LPL-HINDIII 2/2 genotype as an independent risk factor.

### Risk Profile

After single mutations had a relatively low prevalence in our limited patient cohort and CAD is a multifactorial disease we constructed a gene risk profile according to the definitions. It was a combination of the alleles with the highest incidence of either one of clinical endpoints: Included was hetero- or homozygous a-allele of ***eNOS 4ab ***polymorphism, because patients showed a significantly higher risk to get a rentervention or recurrent symptoms. Regarding the ***ApoE ***they had to carry ε4 allele, that means ε2/4 or ε4/4, as it is considered to be potentielly atherogenous, was we could also demonstrate. Homozygous carriers of hepatic lipase were prone to undergo a reoperation and recurrent symptoms. That's why part of the risk gene profile was homozygous expression of the G allele. Homozygous expression of CETP gene polymorphism was included. Additionally, hetero- or homozygous variants of G20210A mutation was chosen, because only 50 and 0% carriers of these variants were free from recurrent symptoms after 10 and 15 years, respectively. However, a simple gene risk profile constructed out of 7 randomly chosen polymorphisms was more predictive for the advancement of CAD than any cluster of classical risk factors.

Certainly medical therapy, particularly lowering of low-density lipoprotein cholesterol levels, has been proven to reduce the advancement of CAD after CABG [20]. Due to the design of our retrospective study, we were unable to prove the beneficial effects of medical therapy, because therapy after primary CABG was determined by the cardiologist and patients with recurrent symptoms received intensified medical therapy. In the investigated patient's cohort 87% received Aspirine, 70% were given beta blockers, 82% of patients received statins, 35% ACE inhibitors and 24% took calcium antagonists postoperatively. Likewise, the role of classical risk factors on the CAD progression is difficult to interpret. Risk factors at the time of primary CABG were medically treated as described above; patients stopped smoking and started training programs. Therefore, these classical risk factors lost partially their predictive value. Obviously, we investigated only long-term survivors of CABG surgery, who were willing to cooperate. This selection bias may also limit our results.

Knowledge of gene polymorphisms in the era of genomics and their influence on outcome in cardiac surgery is rapidly growing. However, most studies investigated the acute effects of polymorphisms outcome during the postoperative phase. Data on the progression of CAD after primary CABG is rare.

This study is only preliminary, because of its limitations in patient sample size as well as number and choice of investigated polymorphisms. More investigations are warranted and will most likely improve the predictive value of polymorphism tests. We proved the concept, that risk stratification by a simple gene test for the future advancement of CAD after primary CABG is possible. The concept is intriguing, because the detected gene variants give clues to the individual pathophysiology in every single patient in this multifactorial disease [18]. Therefore, this cheap diagnostic tool may hopefully lead to an individualized secondary prevention after primary CABG.

## Conclusion

Classical preoperative risk factors provide little information on appearance of cardiac adverse events, probably because they are so very common among CABG patients. Additionally, most patients were medically treated. Single gene polymorphisms of patients, in the era of whole genome scans, allow a limited prognosis for CAD progression after primary CABG. Risk gene profiles enable risk stratification of CAD progression. They may have the potential to individualize therapy in the future, due to pathophysiological links. Further gene polymorphisms have to be investigated to improve risk stratification.

## Competing interests

The authors declare that they have no competing interests. Institutional review board approval was received before investigations have been started.

## Authors' contributions

SE, AR, GN, BR, PL have made substantial contributions to conception and design, or acquisition of data, or molecular genetic analysis and interpretation of data; SE, ABF and PL have been involved in drafting the manuscript or revising it critically for important intellectual content; and all authors have read and given final approval of the version to be published.

## Supplementary Material

Additional file 1 **Investigated genes, their polymorphisms and function, primer and primer sequences**. Polymorphisms in genes coding for apolipoprotein E (APOE) [4,8], hepatic lipase (LIPC), cholesteryl ester transfer protein (CETP), endothelial NO synthase (NOS3), and plasminogen activator inhibitor 1 (SERPINE1), coagulation factor V (F5), and coagulation factor II (prothrombin (F2)) were chosen. Details of the polymorphisms are summarized in this additional file 1. Abbreviations: A: Arginin; C: Cytosin; G: Guanin; T: Thymin; HDL: high density lipoprotein; LDL: low density lipoprotein; VLDL: very low density lipoprotein Click here for file
