# Supplementary material for: Gene polymorphisms in APOE, NOS3, and LIPC genes may be risk factors for cardiac adverse events after primary CABG
Source: J Cardiothorac Surg. 2009 Aug 19;4:46. doi: 10.1186/1749-8090-4-46 (PMC2736166; doi:10.1186/1749-8090-4-46)
Supplement: Additional file 1 — Investigated genes, their polymorphisms and function, primer and primer sequences. Polymorphisms in genes coding for apolipoprotein E (APOE) [4,8], hepatic lipase (LIPC), cholesteryl ester transfer protein (CETP), endothelial NO synthase (NOS3), and plasminogen activator inhibitor 1 (SERPINE1), coagulation factor V (F5), and coagulation factor II (prothrombin (F2)) were chosen. Details of the polymorphisms are summarized in this additional file 1. Abbreviations: A: Arginin; C: Cytosin; G: Guanin; T: Thymin; HDL: high density lipoprotein; LDL: low density lipoprotein; VLDL: very low density lipoprotein [file 1749-8090-4-46-S1.ppt]

## Slide 1
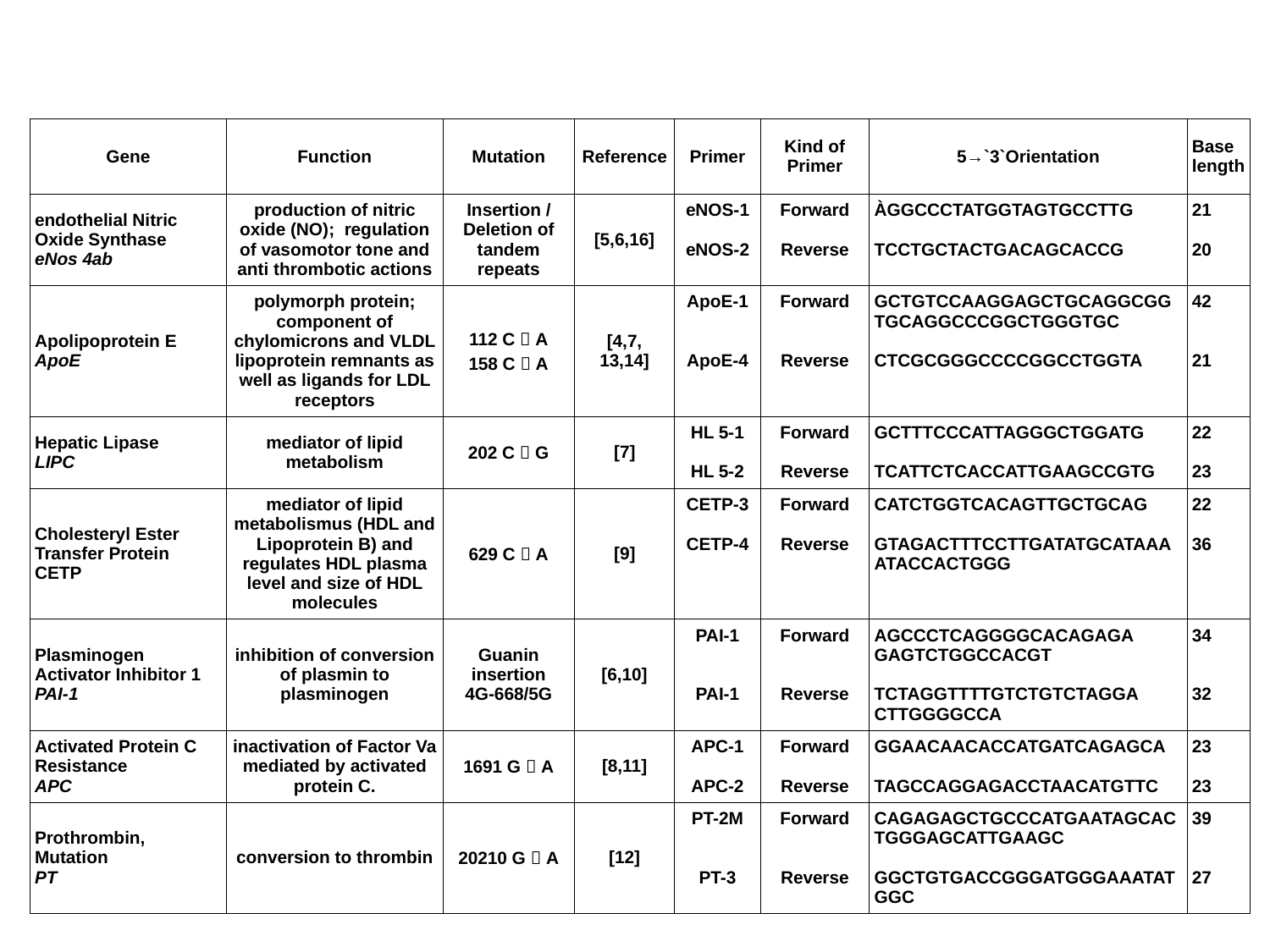

| Gene | Function | Mutation | Reference | Primer | Kind of Primer | 5→`3`Orientation | Base length |
| --- | --- | --- | --- | --- | --- | --- | --- |
| endothelial Nitric Oxide Synthase eNos 4ab | production of nitric oxide (NO); regulation of vasomotor tone and anti thrombotic actions | Insertion / Deletion of tandem repeats | [5,6,16] | eNOS-1 eNOS-2 | Forward Reverse | ÀGGCCCTATGGTAGTGCCTTG TCCTGCTACTGACAGCACCG | 21 20 |
| Apolipoprotein E ApoE | polymorph protein; component of chylomicrons and VLDL lipoprotein remnants as well as ligands for LDL receptors | 112 C  A 158 C  A | [4,7, 13,14] | ApoE-1 ApoE-4 | Forward Reverse | GCTGTCCAAGGAGCTGCAGGCGGTGCAGGCCCGGCTGGGTGC CTCGCGGGCCCCGGCCTGGTA | 42 21 |
| Hepatic Lipase LIPC | mediator of lipid metabolism | 202 C  G | [7] | HL 5-1 HL 5-2 | Forward Reverse | GCTTTCCCATTAGGGCTGGATG TCATTCTCACCATTGAAGCCGTG | 22 23 |
| Cholesteryl Ester Transfer Protein CETP | mediator of lipid metabolismus (HDL and Lipoprotein B) and regulates HDL plasma level and size of HDL molecules | 629 C  A | [9] | CETP-3 CETP-4 | Forward Reverse | CATCTGGTCACAGTTGCTGCAG GTAGACTTTCCTTGATATGCATAAAATACCACTGGG | 22 36 |
| Plasminogen Activator Inhibitor 1 PAI-1 | inhibition of conversion of plasmin to plasminogen | Guanin insertion 4G-668/5G | [6,10] | PAI-1 PAI-1 | Forward Reverse | AGCCCTCAGGGGCACAGAGA GAGTCTGGCCACGT TCTAGGTTTTGTCTGTCTAGGA CTTGGGGCCA | 34 32 |
| Activated Protein C Resistance APC | inactivation of Factor Va mediated by activated protein C. | 1691 G  A | [8,11] | APC-1 APC-2 | Forward Reverse | GGAACAACACCATGATCAGAGCA TAGCCAGGAGACCTAACATGTTC | 23 23 |
| Prothrombin, Mutation PT | conversion to thrombin | 20210 G  A | [12] | PT-2M PT-3 | Forward Reverse | CAGAGAGCTGCCCATGAATAGCACTGGGAGCATTGAAGC GGCTGTGACCGGGATGGGAAATATGGC | 39 27 |
